# Adolescents and young adults with germline *CDH1* variants and the risk of overtreatment

**DOI:** 10.1093/jnci/djaf002

**Published:** 2025-01-06

**Authors:** Amber F Gallanis, Cassidy Bowden, Rachael Lopez, Grace-Ann Fasaye, David Lang, Jill Rothschild, M Constanza Camargo, Jonathan M Hernandez, Anjali Rai, Theo Heller, Andrew M Blakely, Jeremy L Davis

**Affiliations:** Center for Cancer Research, National Cancer Institute, National Institutes of Health, Bethesda, MD 20892, United States; Center for Cancer Research, National Cancer Institute, National Institutes of Health, Bethesda, MD 20892, United States; Clinical Center Nutrition Department, National Institutes of Health, Bethesda, MD 20892, United States; Genetics Branch, National Cancer Institute, National Institutes of Health, Bethesda, MD 20892, United States; Department of Pediatrics, National Institutes of Health Clinical Center, Bethesda, MD 20892, United States; Office of Patient Safety and Clinical Quality, National Institutes of Health Clinical Center, Bethesda, MD 20892, United States; Department of Pediatrics, National Institutes of Health Clinical Center, Bethesda, MD 20892, United States; Division of Cancer Epidemiology and Genetics, National Cancer Institute, National Institutes of Health, Bethesda, MD 20892, United States; Center for Cancer Research, National Cancer Institute, National Institutes of Health, Bethesda, MD 20892, United States; National Institute of Diabetes and Digestive and Kidney Diseases, National Institutes of Health, Bethesda, MD 20892, United States; National Institute of Diabetes and Digestive and Kidney Diseases, National Institutes of Health, Bethesda, MD 20892, United States; Center for Cancer Research, National Cancer Institute, National Institutes of Health, Bethesda, MD 20892, United States; Center for Cancer Research, National Cancer Institute, National Institutes of Health, Bethesda, MD 20892, United States

## Abstract

**Background:**

Adolescents and young adults (AYA) with germline *CDH1* variants are at risk of overtreatment when precancer lesions are detected with endoscopic screening. We characterize diffuse-type gastric cancer prevalence and survival in AYA managed with prophylactic total gastrectomy (PTG) or endoscopic surveillance.

**Methods:**

Prospective cohort study of 188 individuals aged 39 and younger enrolled from January 27, 2017, to May 1, 2023. Clinicopathological data, prevalence of early gastric signet ring cell (SRC) lesions, advanced gastric cancer diagnoses, and cancer-specific survival were measured.

**Results:**

Among 188 AYA patients, 104 chose surveillance and 67 pursued PTG for management of elevated gastric cancer risk. AYA who enrolled early in the study period and had SRC lesions detected on preoperative endoscopy were more likely to elect for PTG compared with surveillance. SRC lesions were detected on preoperative endoscopy in 48% of patients who subsequently had PTG, and yet nearly all (93%, 62/67) had multifocal SRC (pT1aN0) on final pathology. Median age at enrollment (30 vs 31 years, *P* = .21), biological sex (*P* = .17), and median number of family members with gastric cancer (3 vs 4, *P* = .14) were not different between groups. No patients under surveillance developed advanced cancer or developed cancer recurrence after PTG with a median follow-up of 2.5 years (IQR = 1.6-4.0) from initial endoscopy.

**Conclusions:**

Cancer-specific outcomes were not different in AYA who harbored SRC and were managed with surveillance or PTG. Lack of cancer-specific deaths and low prevalence of advanced gastric cancer underscore the risk of overtreatment of SRC lesions and suggest that active surveillance is warranted.

## Introduction

Gastric adenocarcinoma is rare in pediatric and young adult populations.^1^^,^^2^ However, in the United States the incidence of gastric cancer in individuals aged 39 and younger increased by 4%-5% overall from 1990 to 2019.^3^ Heritable cancer syndromes are more frequently reported as the cause of gastric cancer in children than adults.^4^ Hereditary diffuse gastric and lobular breast cancer syndrome is characterized by early onset of diffuse-type cancers with signet ring cell (SRC) features and lobular breast cancer.^5-7^ The most frequent cause is loss-of-function mutations in the *CDH1* tumor suppression gene, which encodes the cell-cell adhesion protein, E-cadherin.^8^ Germline testing for *CDH1* variants, and more recently *CTNNA1*, is offered at the age of consent for individuals who meet testing criteria.^5^ Current consensus guidelines recommend prophylactic total gastrectomy (PTG) at age 20-30 years for individuals with germline *CDH1* pathogenic and likely pathogenic (P/LP) variants and detection of intramucosal SRC lesions by endoscopy.^5^

Assessment of gastric cancer risk due to *CDH1* P/LP variants is complex, and the onus is on clinicians to correctly interpret individual cancer risk, because risks of both missed diagnoses and overtreatment can be severe.^9^^,^^10^ Prophylactic surgery is accepted in cancer syndromes such as multiple endocrine neoplasia type 2, for which prophylactic thyroidectomy is endorsed universally; however, a dearth of evidence in adolescents and young adults (AYA) with *CDH1* variants can lead to inconsistent clinical management.^11^ Currently, there are only case reports of adolescent children who underwent PTG, and guidance specific to AYA with gastric cancer is limited.^12-14^ Active surveillance with upper endoscopy is an alternative management strategy to prophylactic surgery in adults and has demonstrated safety and efficacy in single-institution cohort studies.^15^^,^^16^ Even so, microscopic SRC lesions are commonly found in *CDH1* P/LP variant carriers and have been reported in asymptomatic individuals as young as 10 years of age.^14^ SRC lesions are ubiquitous in *CDH1* P/LP variant carriers, and yet lifetime advanced gastric cancer risk may be as low as 7%-10% in individuals with no family history of gastric cancer.^17^ Emerging data suggest that individuals with *CDH1* P/LP variants under endoscopic surveillance rarely develop greater than stage pT1a gastric carcinoma, although studies with long-term follow-up are lacking.^18^ Despite this, detection of SRC lesions during endoscopy in AYA often results in a recommendation for PTG.^5^ Because the physical and psychological sequelae of PTG are life-altering, it is imperative to understand the natural history of SRC lesions that arise in the context of germline *CDH1* P/LP variants.^9^ The primary aim of this study was to characterize gastric cancer-specific outcomes among AYA with *CDH1* P/LP variants who were managed with PTG or endoscopic surveillance. We explored the prevalence of gastric cancers among AYA and their family members and examined factors associated with age at gastric cancer diagnosis. Last, we provide a framework to inform clinical decision-making for AYA at risk for hereditary diffuse-type gastric cancer.

## Methods

### Study design

AYA, defined as individuals aged 39 years and younger, enrolled in a prospective, single-institution study of hereditary gastric cancer (NCT03030404) between January 27, 2017, and May 1, 2023, were included in this analysis. This study was approved by the institutional review board, and all patients completed informed written consent. Minor patients provided assent for the study and surgery, with written consent provided by parents or legal guardians. All participants underwent germline genetic testing at clinical diagnostic laboratories before study enrollment, and genetic test reports were reviewed by a genetic counselor and the principal investigator. Individuals with *CDH1* variants of unknown significance and *CTNNA1* variants were excluded. Pathogenicity of each *CDH1* variant was confirmed manually using publicly available data curation in ClinVar and/or in consultation with the ClinGen *CDH1* variant curation expert panel, which includes 2 study authors (G.F. and J.L.D.).^19^ Asymptomatic patients with *CDH1* P/LP variants were counseled according to consensus guidelines.^5^ Prophylactic total gastrectomy was defined as surgery performed in asymptomatic individuals with no gross mucosal abnormalities of the stomach noted on preoperative endoscopy. All gastrectomy operations were performed by a single surgeon via an open approach with a Roux-en-Y esophagojejunostomy and perigastric (D1) lymphadenectomy. Endoscopic surveillance was offered as an alternative to PTG for those who wished to delay surgery or were medically unfit for surgery. Endoscopic surveillance was performed as reported previously by expert gastroenterologists, including a specialist in pediatric gastroenterology.^15^^,^^16^ Two methods of systematic gastric mapping were used in this study. The Cambridge method included 5 nontargeted or random biopsies taken from the following 6 anatomic locations: cardia, fundus, body, transitional zone, antrum, and pre-pylorus (30 biopsies total).^16^ The Bethesda method, described previously, included 4 nontargeted biopsies of 22 anatomic sites (88 biopsies total).^15^ Targeted biopsy of abnormal mucosal findings (ie, ulceration, erosions, polyps, pale spots, and nodularity) was performed in both methods of gastric mapping. Gastric biopsy and gastrectomy specimens were reviewed by gastrointestinal pathologists. Standardized perioperative care was provided by a multidisciplinary team, including a surgical oncologist, gastroenterologists, anesthesiologists, social worker, genetic counselor, registered dietitian, psychologist, and pharmacist.^20^ Patients younger than age 18 were evaluated by hospital pediatricians.

### Statistical analysis

Categorical data were expressed as totals and proportions and compared using χ^2^ analyses with Fisher exact test when indicated. Continuous data were expressed as median with IQR and compared using Wilcoxon rank sum tests. Univariable and multivariable logistic regression analyses were performed to identify associations between patient and clinicopathological characteristics and likelihood of undergoing PTG vs surveillance. Receiver operator characteristic curves were constructed to determine if age of AYAs’ relatives at the time of the relative’s cancer diagnosis was associated with the decision of AYA patients to undergo PTG. Bivariate analyses were performed to compare age at cancer diagnosis of AYA vs age of their relatives. Two-sided *P* value <.05 indicated statistical significance. Statistical analyses were performed using GraphPad Prism, version 9.3.1 (GraphPad Software, Inc., San Diego, CA), JMP Statistical software, version 16.2.0 (JMP, Cary, NC), and RStudio, version 2022.07.2 (RStudio, Boston, MA).

## Results

**Figure 1.**
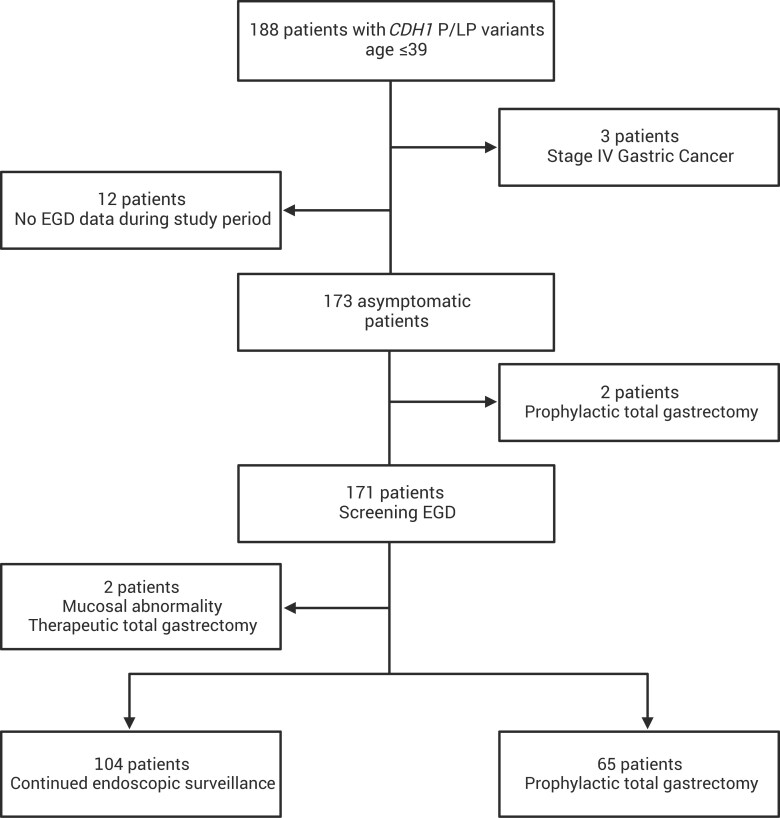
Flowchart of clinical management of adolescents and young adults with *CDH1* P/LP variants. A total of 171 patients underwent screening or baseline endoscopy before electing for endoscopic surveillance or prophylactic total gastrectomy. Abbreviation: EGD = esophagogastroduodenoscopy. Graphical representation created with biorender.com.

One hundred eighty-eight individuals aged 39 years and younger with germline *CDH1* P/LP variants from 132 kindreds were enrolled from January 27, 2017, to May 1, 2023 (Figure 1). The median age at time of germline genetic testing was 28 years (range = 8-39 years), and the median age at study enrollment was 31 years (range = 12-39 years). Most AYA were female (70%, 131/188) and non-Hispanic White (86%, 161/188) (Table 1). Splicing variants (34%, 64/188) were the most frequently observed germline *CDH1* P/LP variants, followed by nonsense (30%, 56/188), frameshift (26%, 49/188), large deletion (7%, 14/188), large duplication (2%, 4/188), and start-loss variants (0.5%, 1/188) (Table S1). Three patients (ages 28-33) had a concurrent diagnosis of metastatic gastric cancer at the time of study enrollment; 2 underwent gastrectomy and cytoreductive surgery with hyperthermic intraperitoneal chemotherapy as a part of a clinical trial, and 1 received palliative systemic chemotherapy.^21^ Two of the 3 individuals had relatives diagnosed with advanced gastric cancer at ages 58 and 47, respectively (Table S2). Nearly all (91%, 171/188) AYA underwent screening endoscopy vs 12 patients (aged 19-37) who had no endoscopy data recorded during the study period and 2 who proceeded to gastrectomy without screening endoscopy (Figure 1). Two individuals (1%, 2/171) had abnormal findings on initial endoscopy: 1 had focal mucosal ulceration that prompted multimodal treatment for pT2N0 gastric cancer (age 33 years), and the other had a focal abnormality with endoscopic ultrasound staging (cT1bN0) that was treated with neoadjuvant chemotherapy and total gastrectomy (ypT1aN0; age 31 years).^22^

**Table 1.** Patient demographics and comparison of adolescents and young adults with germline *CDH1* pathogenic or likely pathogenic variants who elected for endoscopic surveillance vs PTG.

Patient demographics	n = 188
Age at enrollment, median (IQR), years	31 (26-35)
Female, No. (%)	131 (70%)
Male, No. (%)	57 (30%)
***Race***	
White, non-Hispanic	161 (86%)
White, Hispanic	9 (5%)
Hispanic	7 (4%)
Black	6 (3.2%)
Asian	2 (1%)
Multiracial	2 (1%)
American Indian	1 (0.5%)
***CDH1 variants***	
Splicing	64 (34%)
Nonsense	56 (30%)
Frameshift	49 (26%)
Large deletion	14 (7%)
Large duplication	4 (2%)
Start-loss	1 (0.5%)
***Family history***	
FH of gastric cancer	161 (86%)
No. of family members with GC, median (IQR)	3 (2-5)
FH of breast cancer	141 (75%)
No. of family members with BC, median (IQR)	2 (1-3)

	Endoscopic surveillance group n = 104	PTG group n = 67	*P*
Age at enrollment, median (IQR), years	30 (24-34)	31 (27-35)	.21
Female, No. (%)	68 (65%)	51 (76%)	.17
Male, No. (%)	36 (35%)	16 (24%)	
***Enrollment period***			
Early (2/2017-5/2019)	14 (13%)	26 (39%)	.**0002**
Middle (6/2019-5/2021)	36 (35%)	23 (34%)	
Late (6/2021-5/2023)	54 (52%)	18 (27%)	
***Race***			
White, non-Hispanic	86 (83%)	62 (93%)	.18
White, Hispanic	6 (6%)	1 (1%)	
Hispanic	3 (3%)	2 (3%)	
Black	5 (5%)	1 (1%)	
Asian	2 (2%)	0 (0%)	
Multiracial	1 (1%)	1 (1%)	
American Indian	1 (1%)	0 (0%)	
***CDH1 variants***			
Splicing	30 (29%)	27 (40%)	.32
Nonsense	35 (34%)	17 (25%)	
Frameshift	26 (25%)	18 (27%)	
Large deletion	10 (10%)	3 (4%)	
Large duplication	3 (3%)	1 (1%)	
Start-loss	0 (0%)	1 (1%)	
***Family history***			
Family history of GC	85 (82%)	61 (91%)	.10
No. of relatives with GC, median (IQR)	3 (1-5)	4 (2-5)	.14
Family history of BC	81 (78%)	49 (73%)	.51
No. of relatives with BC, median (IQR)	2 (1-3)	2 (1-3)	.40
***Total EGDs performed***	210	81	–
No. of endoscopies per patient, median (IQR)	2 (1-3)	1 (1-1)	**<**.**001**
SRC detected per patient on EGD, No. (%)	37 (36%)	31 (48%)^a^	.15
SRC absent per patient on EGD, No. (%)	67 (64%)	34 (52%)^a^	
***Follow-up from initial EGD, median (IQR), years***	2.1 (1.2-3.2)	3.7 (2.1-4.4)	**<**.**001**
Time from EGD to PTG (SRC detected), median (IQR), months	–	4.5 (3-7.6)	.80
Time from EGD to PTG (SRC absent), median (IQR), months	–	4.4 (1.5-7.4)	
***Age at PTG, median (IQR), years***	–	31 (26-34)	–
SRC present on gastrectomy specimen, No. (%)	–	63 (94%)^b^	–
SRC absent on gastrectomy specimen, No. (%)	–	4 (6%)	–
***Surgical pathology***			
pT0N0, No. (%)	–	4 (6%)	–
pTisN0, No. (%)	–	1 (1%)	–
pT1aN0, No. (%)	–	62 (93%)	–

a n = 65; 2 individuals did not undergo screening endoscopy before PTG.

b Includes 1 patient with pTisN0 disease.

Abbreviations: BC = breast cancer; EGD = esophagogastroduodenoscopy; GC = gastric cancer; IQR = interquartile range 1-3; PTG = prophylactic total gastrectomy; SRC = signet ring cells.

### Endoscopic surveillance cohort

**Figure 2.**
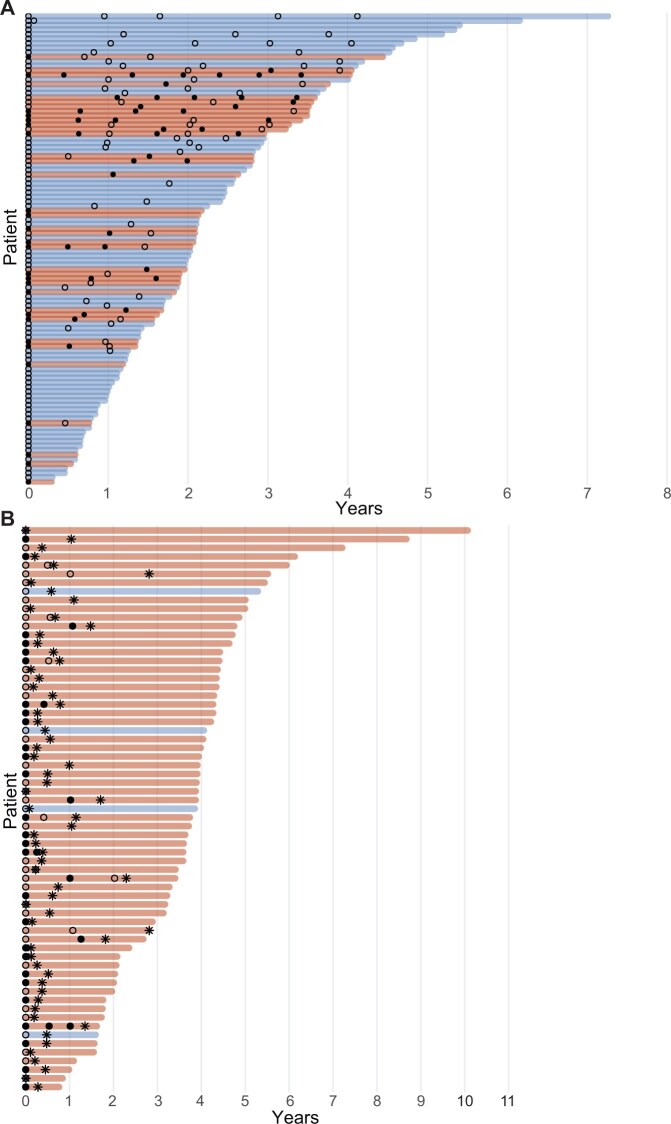
AYA who pursued endoscopic surveillance and prophylactic total gastrectomy. **A**) A total of 104 AYA elected for endoscopic surveillance after screening endoscopy. **Filled and empty circles** represent SRC detected or absent on endoscopic biopsy, respectively. **Red bars** indicate patients who had occult SRC detected on a random biopsy on at least 1 EGD. **Blue bars** are patients in whom no SRC have been detected on endoscopy. **B**) A total of 65 AYA elected for PTG after screening endoscopy. Two patients proceeded immediately to PTG and are not represented here. **Red bars** indicate SRC detected on EGD and/or total gastrectomy specimen. **Filled circles** represent SRC detected on endoscopic biopsy, and **open circles** represent negative biopsies. **Asterisks** represent time point at PTG. Time is represented in years since first EGD. Abbreviations: AYA = adolescents and young adults; EGD = esophagogastroduodenoscopy; PTG = prophylactic total gastrectomy; SRC = signet ring cells.

After *CDH1* diagnosis, 60% (104/173) of asymptomatic AYA chose endoscopic surveillance for management of increased gastric cancer risk. Reluctance to undergo surgery, family planning, and concerns about postgastrectomy lifestyle changes were common reasons for electing surveillance over gastrectomy.^10^ Individuals who elected surveillance had a median age of 30 years (IQR = 24-34) at the time of study enrollment and were mostly female (65%, 68/104) (Table 1). Most patients (82%, 85/104 and 78%, 81/104) reported a family history of gastric and breast cancer, respectively. In total, 210 surveillance endoscopies were performed in the AYA surveillance cohort with a median of 2 endoscopies per patient (IQR = 1-3). No serious adverse events related to surveillance endoscopies were reported. Occult SRC were detected by endoscopic biopsy in 37 (36%) of 104 individuals. Importantly, no patients who were under surveillance developed advanced gastric cancer (AJCC Stage ≥II) with a median time of 2 years (range = 0.3-7.3 years) from initial esophagogastroduodenoscopy (EGD) (Figure 2, A). Median follow-up for individuals was not different based on SRC detection at endoscopy (SRC positive: 2.1 years, range = 0.3-4.4 years; SRC negative: 2.0 years, range = 0.3-7.3 years; *P* = .64).

### Prophylactic total gastrectomy cohort

Sixty-seven (39%, 67/173) individuals elected for PTG, 2 of whom proceeded to surgery without preoperative endoscopy. The median age at the time of operation was 31 years (IQR = 26-34); most patients were women (76%, 51/67) and reported a family history of gastric cancer (91%, 61/67) and breast cancer (73%, 49/67) (Table 1). Thirty-day hospital readmission and reintervention rates were low (6%, 3/52 and 8%, 4/52, respectively; Table S3).^9^ Nearly all (93%, 62/67) PTG specimens harbored occult SRC lesions (pT1aN0, Stage IA) compared with 48% (31/65) SRC detection rate on preoperative endoscopy. Thirty-one individuals (48%, 31/65) had a preoperative endoscopy on which SRC lesions were detected (Figure 2, B). Thus, the false negative detection rate of SRC lesions was 49%, which is consistent with prior reports.^23^ Less frequent pathological diagnoses included 1 patient (1%, 1/67) with carcinoma in situ and 4 patients (6%, 4/67) who had no cancer detected on gastrectomy specimen. Length of time from EGD to PTG was not different based on endoscopic detection of SRC lesions (SRC positive: 4.5 months, IQR = 3-7.6; SRC negative: 4.4 months, IQR = 1.5-7.4; *P* = .80) (Figure 2, B). With a median follow-up of 3.3 years (IQR = 1.7-4) from the date of PTG, there were no cancer-related deaths or gastric cancer recurrences.

The number of AYA who elected for PTG vs endoscopic surveillance varied by enrollment period. AYA who enrolled later in the study period (6/2019-5/2023) were more likely to elect for surveillance compared with PTG (*P* < .0001), likely reflecting changes in clinical experience (Table S4).^15^ AYA who elected for gastrectomy were more likely to have SRC detected on preoperative endoscopy (*P* = .025). There was no difference in family history of gastric cancer (91% and 82%, *P* = .10) and *CDH1* variant type (*P* = .32) between those who had PTG and surveillance, respectively (Table 1). There were no differences in median age at enrollment (30 years, IQR = 24-35 vs 31 years, IQR = 27-35, *P* = .21), biological sex (*P* = .17), race/ethnicity (*P* = .18), median number of family members with gastric cancer (3 vs 4, *P* = .14), or breast cancer (2 vs 2, *P* = .40) among individuals who elected for surveillance and PTG, respectively (Table 1). Median follow-up from initial EGD was longer in the PTG group (3.7 years, IQR = 2.1-4.4) than the surveillance group (2.1 years, IQR = 1.2-3.2; *P* < .001).

### Prevalence of gastric cancer in AYA families

**Figure 3.**
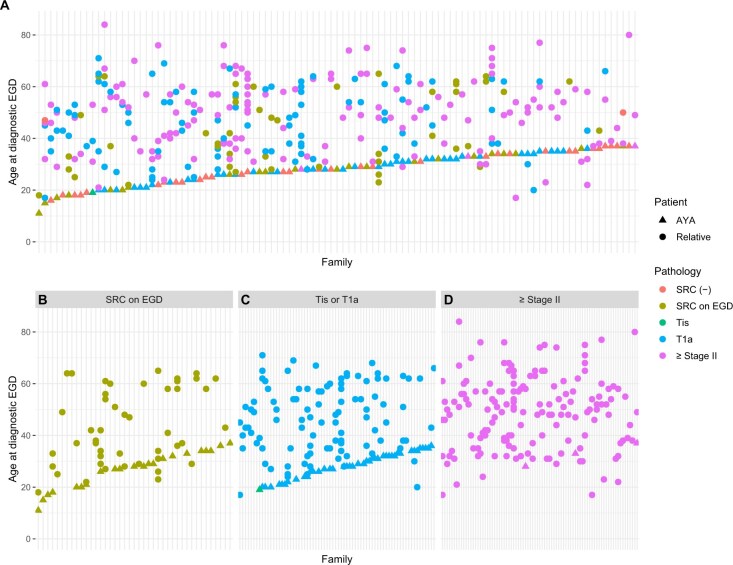
Distribution of gastric SRC and advanced gastric cancer diagnoses by age. **A**) Detection of SRC in AYA (**green, blue, and purple triangles**) by increasing age of AYA cancer diagnosis or age at first endoscopy where no SRC were detected (**red triangles**). Disease stage and age of cancer diagnosis in AYA relatives (**circles**) for 101 kindreds. **B**) **Green** indicates occult SRC detected on EGD, (**C**) **blue** indicates pT1aN0 and **teal** indicates pTis disease demonstrated on PTG specimen, and (**D**) **purple** indicates advanced (≥Stage II) gastric cancer. Thirty-three AYA had family members with gastric cancer and no personal history of SRC on EGD (**red triangles**). Abbreviations: AYA = adolescents and young adults; EGD = esophagogastroduodenoscopy; PTG = prophylactic total gastrectomy; SRC = signet ring cell.

AYA frequently reported a family history of gastric cancer (86%, 161/188) and breast cancer (75%, 141/188). Prior studies have suggested that gastric cancer risk is cumulative with age and that an optimal age exists before which PTG should be performed.^24^ To address this, we examined detailed pedigrees with family cancer histories of at least 3 generations that were available for 101 of 132 kindreds (Figure 3, A). One in 4 families (27%, 27/101) had at least 1 member who had SRC detected by endoscopy with biopsies (Figure 3, B). We identified that more than half (52%, 53/101) of kindreds had at least 1 family member who underwent PTG that harbored occult SRC lesions (pT1aN0) (Figure 3, C). Most kindreds (82%, 83/101) had at least 1 family member who was diagnosed with advanced (Stage ≥II) gastric cancer (Figure 3, D). Among family members of AYA, age at diagnosis of SRC by endoscopy was younger than age at diagnosis of advanced gastric cancer (Stage ≥II) (41 vs 51 years, respectively; *P* = .02). This may be attributed to cascade genetic testing. However, there was no difference in median age at which family members were diagnosed with SRC via endoscopy (41 years, range = 18-65 years) or at time of PTG (47 years, range = 17-71 years; *P* = .37).

To understand the age-related distribution of diffuse gastric cancer risk, we compared age of cancer diagnosis in AYA and affected family members according to findings of SRC on endoscopy, Stage IA gastric cancer (at PTG), and advanced (Stage ≥II) gastric cancer (Figure 3). We found that increasing age of AYA diagnosed with SRC on endoscopy was positively correlated with increasing age of family member cancer diagnosis, irrespective of family member cancer stage (*P* = .0034) (Figure S1). Individuals with *CDH1* and a family history of gastric cancer are presumably more likely to have a screening endoscopy at a younger age because of cascade genetic testing. There was no association between family member cancer diagnosis and age of cancer diagnosis in AYA patients with Stage IA SRC (*P* = .96) or advanced gastric cancer (Stage ≥II) (*P* = .43). There was no association between a relative’s gastric cancer stage and AYA odds of undergoing gastrectomy (*P* = .81, Table S4). One-third (33/101) of AYA patients did not have SRC detected on endoscopy despite having multiple family members diagnosed with gastric cancer (Figure 3, A).^15^^,^^16^ Taken together, these data suggest that there is no peak age at which gastric cancer affects families with *CDH1* variants.

## Discussion

We report no cancer deaths and no cases of advanced gastric cancer in AYA who underwent active surveillance instead of PTG for management of germline *CDH1* P/LP variants. Notably, these outcomes were observed in individuals who had SRC detected by endoscopy many years before and were irrespective of whether individuals proceeded to PTG. We showed that occult SRC lesions were present in nearly all patients (93%) who underwent PTG even though SRC were detected in less than half of preoperative endoscopies; these findings were irrespective of age, biological sex, and *CDH1* variant type. Our results reinforce previous reports of the ubiquity and likely premalignant nature of SRC among *CDH1* P/LP variant carriers.^25^ Previous studies suggested an optimal age for PTG;^24^ however, these studies likely cause undue distress for patients who are diagnosed at an age older than the purported optimal age for PTG. Last, we showed that there was no peak age or familial pattern for age of onset of advanced gastric cancer. The data presented here reinforce previous work that SRC are an expected finding in germline *CDH1* P/LP variant carriers and do not herald clinically actionable cancer.^25^^,^^26^

Individuals with *CDH1* P/LP variants may assume their individual cancer risk mirrors that of affected family members; however, our results do not support a familial pattern of age of onset of advanced gastric cancer. We showed that AYA are often diagnosed with SRC lesions by EGD at an earlier age than their relatives; however, this may be attributed to greater participation in cascade genetic testing. It is important to view these data with respect to emerging evidence on cumulative advanced gastric cancer risk that is lower than previously described, especially in families with no known history of gastric cancer.^27^ However, providers should consider family history of cancer in *CDH1* P/LP variant carriers because a strong family history of gastric cancer, defined as 3 first-degree relatives with gastric cancer, is associated with a higher estimated lifetime risk of gastric cancer of 38%.^27^ Although family history should be considered when assessing lifetime cancer risk, the age at diagnosis of advanced gastric cancer within a family can be variable and is likely governed by other, as yet unknown, factors. The authors caution against overtreatment of occult SRC lesions, particularly in individuals with no family history of gastric cancer, because SRC appear to be indolent precursors of gastric cancer. Additionally, clinical experience with PTG in pediatric patients with *CDH1* variants is limited.^12-14^ Thus, case reports of SRC detection by endoscopy or diagnosis of Stage IA gastric cancer in children with *CDH1* variants should be interpreted with caution.

Gastric cancer screening is recommended after a diagnosis of a germline *CDH1* P/LP variant because incident gastric cancers, although uncommon, have been detected at initial endoscopy.^5^ We described one individual with an ulcerated lesion detected at screening endoscopy, which resulted in multimodality treatment of a Stage IB gastric cancer. We have previously demonstrated that gross mucosal abnormalities that harbor SRC on biopsy should prompt transition from surveillance to therapeutic intervention.^15^ However, in the setting of SRC detected on nontargeted biopsies, we have reported that endoscopic surveillance can be a safe alternative to PTG.^15^ We have shown that when SRC lesions are detected on random biopsy, in the setting of normal-appearing gastric mucosa, the most advanced pathological finding in the total gastrectomy explant is pT1aN0 gastric carcinoma.^15^ Furthermore, the use of systemic chemotherapy for occult, Stage IA gastric cancer is likely unnecessary.^28^ Turgeon and colleagues demonstrated surgery alone for clinical Stage I SRC gastric cancer was associated with better 5-year overall survival compared with those who received neoadjuvant and adjuvant chemotherapy.^28^ Similarly, Li et al. observed no survival benefit associated with neoadjuvant chemotherapy compared with surgery alone in patients with resectable SRC.^29^ Therefore, we caution the use of perioperative chemotherapy for individuals with germline *CDH1* P/LP variants and SRC given the absence of a survival benefit.^30^^,^^31^

AYA are tasked with incredibly difficult and potentially life-changing decisions related to management of their germline *CDH1* variants. The data presented here illustrate the sort of cognitive dissonance that arises when occult SRC lesions are classified as pathological Stage IA cancers. Even so, the indolent nature of SRC lesions arising due to germline *CDH1* P/LP variants strongly suggests that these individuals should not be treated the same as those with sporadic (nonsyndromic) gastric cancers. In the absence of abnormal endoscopic findings (eg, mucosal ulceration), we suggest surveillance as an alternative to PTG to individuals, especially in minors, who have SRC detected by endoscopy, irrespective of SRC focus size (Table 2).^15^ Even though 8% (15/188) of our cohort underwent genetic testing at age 17 or younger, the authors support genetic testing of those at risk of hereditary diffuse gastric cancer starting at age 18, followed by initial screening endoscopy. For patients younger than age 18, endoscopy should be considered if the patient endorses unexplained gastrointestinal symptoms. The current study provides much needed data on this cancer predisposition syndrome and warrants the attention of clinicians who care for AYA, in whom germline genetic testing has become widely available. Our study is limited by disease rarity and median overall follow-up of 2.5 years. However, we reported no gastric cancer recurrences during this period, when individuals with gastric cancer are at highest risk of cancer recurrence.^32^ Also, the findings from our North American study cohort may not be applicable to well-defined ancestral groups with high disease penetrance in other regions of the world. Additional studies with long-term follow-up are warranted in individuals with germline *CDH1* variants who elect for endoscopic surveillance over PTG.

**Table 2.** Proposed management of adolescents and young adults (AYA) with germline *CDH1* pathogenic or likely pathogenic variants.

Guidelines for AYA at risk of hereditary diffuse gastric cancer
Genetic testing Age 18 years or older; parents may consider testing minor children based on family history of advanced gastric cancer and/or maturity level of minor
Screening endoscopy At initial diagnosis of *CDH1* P/LP variant if age 18 or older If younger than age 18, consider initial endoscopy if unexplained gastrointestinal symptoms
Management of (+) signet ring cell If positive biopsy is from a gross mucosal abnormality, such as an ulcerated lesion, proceed to workup and treatment for advanced diffuse gastric cancer If normal mucosa and biopsy was obtained randomly, then repeat endoscopy in 6 months, especially in patients younger than age 18 If patient age is younger than 18, refer to center of excellence for a multidisciplinary assessment including a surgical oncologist, gastroenterologist, pediatrician, dietitian, social worker, genetic counselor, and psychologist

## Conclusions

Cancer-specific outcomes in AYA with germline *CDH1* P/LP variants who pursue active surveillance are no different than those who had PTG. The prevalence of advanced gastric cancers is low in this population even though SRC lesions are frequent, which further supports their designation as precancers.^25^ In the context of a germline *CDH1* variant, AYA with occult SRC lesions detected by endoscopic surveillance are at risk of overtreatment with gastrectomy. Ongoing research aimed at identifying factors that govern the progression of SRC precursors to advanced diffuse-type gastric cancer is critically important. The data presented here can help clinicians and patients make informed decisions regarding gastric cancer risk management. Young individuals with germline *CDH1* P/LP variants and gastric cancer warrant specialty care in expert centers, ideally in the context of ongoing clinical research.

## Supplementary Material

djaf002_Supplementary_Data

## Data Availability

Deidentified data may be shared upon reasonable request from the corresponding author.
