# Supplementary material for: Adolescents and young adults with germline CDH1 variants and the risk of overtreatment
Source: J Natl Cancer Inst. 2025 Jan 6;117(5):1027–35. doi: 10.1093/jnci/djaf002 (PMC12058253; doi:10.1093/jnci/djaf002)

Supplementary Material

Supplementary Tables

Supplementary Table 1. Adolescents and young adults with germline *CDH1* P/LP variant classification and type.

# AYA with variant (n)	*CDH1* P/LP variant	Variant type	# Families with each variant (n)	Self- reported ancestry
1	c.1064dup	Frameshift	1	European–English
1	c.1131delC	Frameshift	1	European
1	c.1145del	Frameshift	1	European–English
1	c.1212del*	Frameshift	1	European–Irish
1	c.124_126delinsT*	Frameshift	1	European–Dutch, Irish
1	c.1341del*	Frameshift	1	European–Croatian, Danish
2	c.1342dup	Frameshift	1	F1(2): African American
2	c.1373del	Frameshift	1	F1(2): European–Irish
1	c.1397_1398del	Frameshift	1	European–Spanish, Irish
2	c.1476_1477del*	Frameshift	1	F1(2): European–German
4	c.1587dup*	Frameshift	2	F1(3): European–English, Irish<br>F2(1): European
1	c.1650del*	Frameshift	1	European
1	c.1779dupC*	Frameshift	1	European
2	c.1895_1896del*	Frameshift	2	F1(1): European–German<br>F2(1): European
1	c.1920dup*	Frameshift	1	European–German, Irish, Polish, Uranian, and Native American
1	c.1999del	Frameshift	1	European–English, Irish
2	c.2054dup*	Frameshift	2	F1(1): European–Norwegian, British<br>F2(1): European
1	c.2276del*	Frameshift	1	European–Italian
1	c.2324del*	Frameshift	1	European–German, Belgium, France, Prussia, Ashkenazi Jewish
1	c.2430del*	Frameshift	1	Mexican, European–Irish
5	c.2474dup*	Frameshift	3	F1(3): European<br>F2(1): European<br>F3(1): European–English, Irish
2	c.261del*	Frameshift	1	F1(2): European
1	c.377del	Frameshift	1	Chinese
1	c.377dup*	Frameshift	1	European–English, French, Irish

2	c.382del	Frameshift	2	F1(1): European–Italian<br>F2(1): European–Irish, Italian
2	c.480_486del*	Frameshift	1	F1(2): European–Swedish, German, English
1	c.504del*	Frameshift	1	European–German
1	c.521dup*	Frameshift	1	European–Polish
2	c.603del*	Frameshift	2	F1(1): European–Hungarian, German<br>F2(1): European–German
1	c.720del*	Frameshift	1	European–Irish, French
1	c.776del*	Frameshift	1	Turkish
2	c.885del	Frameshift	1	F1(2): Middle Eastern–Palestinian
3	Deletion entire gene*	Large Deletion	2	F1(2): European–Scandinavian<br>F2(1): European–German
2	Deletion exon 16*	Large Deletion	2	F1(1): European<br>F2(1): European–German, Irish, English
2	Deletion exon 3*	Large Deletion	2	F1(1): European–Scottish, Irish<br>F2(1): European
6	Deletion exons 1-2*	Large Deletion	6	F1(1): European<br>F2(1): European<br>F3(1): European–Norwegian, English, Greek<br>F4(1): European– German, French, and Native American<br>F5(1): African American<br>F6(1): Peruvian
1	Deletion exons 3-5*	Large Deletion	1	European
4	Gain exons 3-9	Large Duplication	1	F1(4): European–Scandinavian
1	c.1003C>T*	Nonsense	1	European
1	c.1155dup*	Nonsense	1	European
1	c.1189A>T*	Nonsense	1	European
1	c.1227G>A	Nonsense	1	European–German
4	c.172G>T*	Nonsense	1	F1(4): European–Nordic
10	c.1792C>T*	Nonsense	8	F1(3): European<br>F2(1): European–German, English, Norwegian, Swedish, Irish<br>F3(1): European–Irish<br>F4(1): European<br>F5(1): European–English, French, German<br>F6(1): European<br>F7(1): European<br>F8(1): European–Scandinavian
6	c.187C>T*	Nonsense	2	F1(4): Mexican

				F2(2): European–English, Irish, German
20	c.2064_2065del*	Nonsense	12	F1(4): European–Irish, Scottish, German, English<br>F2(3): European–German, French<br>F3(3): European–German, English, Polish<br>F4(2): European<br>F5(1): European–English, Irish, Scottish<br>F6(1): European<br>F7(1): European<br>F8(1): European<br>F9(1): European<br>F10(1): European–English, Irish, Scottish, Swiss, German, French Canadian<br>F11(1): European<br>F12(1): European
5	c.2287G>T*	Nonsense	4	F1(2): European<br>F2(1): European–English, German<br>F3(1): European–Scandinavian<br>F4(1): European–German and English
2	c.2446A>T	Nonsense	1	F1(2): Hispanic–Dominican Republic
3	c.26C>A*	Nonsense	1	F1(3): European–German, French
1	c.385C>T*	Nonsense	1	European–English
1	c.76G>T*	Nonsense	1	European–Swedish
1	c.1137+1G>A	Splicing	1	African American
1	c.1137+2T>C*	Splicing	1	European
2	c.1533_1565+39del*	Splicing	1	F1(2): European–Swedish
4	c.1565+1G>A*	Splicing	4	F1(1): American Indian, Mexican, Central American and European–English, German, Irish, Spanish, Portuguese<br>F2(1): European–Polish<br>F3(1): European–English<br>F4(1): European–Swedish, Danish
3	c.1565+1G>C*	Splicing	1	F1(3): European–Italian
2	c.1565+1G>T*	Splicing	2	F1(1): European–English, Irish<br>F2(1): Unknown
1	c.1565+2_1565+3insTT*	Splicing	1	European–Russian
1	c.1565+5G>A*	Splicing	1	Chinese
3	c.1565+672_1566-23del2827*	Splicing	2	F1(2): European–Italian

				F2(1): European–Italian, French Canadian
2	c.1566-2A>G	Splicing	1	F1(2): European–German
1	c.1711+1G>A*	Splicing	1	European
3	c.1711+2_1711+7del*	Splicing	1	F1(3): European–Swedish, Norwegian, French
2	c.1711G>A*	Splicing	1	F1(2): European–Irish, Italian
1	c.2165-1G>A	Splicing	1	European–Scottish, Irish
6	c.2161-1G>C*	Splicing	1	F1(6): European–German
6	c.2195G>A*	Splicing	4	F1(2): European–Irish, Scottish<br>F2(2): European–German, Polish<br>F3(1): Ecuador<br>F4(1): European– Italian
1	c.49-2A>C*	Splicing	1	European–British Isles
2	c.532-1G>C*	Splicing	2	F1(1): African American<br>F2(1): European
1	c.832+1_832+2delinsTC	Splicing	1	African American
4	c.833-2A>G*	Splicing	4	F1(1): European–English, German<br>F2(1): European–German, English, Austrian<br>F3(1): European<br>F4(1): European
3	c.832+1G>A*	Splicing	2	F1(2): European<br>F2(1): European–Italian
1	c.1008G>T	Splicing	1	European–Scottish
2	c.1137G>A	Splicing	2	F1(1): European<br>F2(1): European–German, Irish, Swedish
11	c.715G>A*	Splicing	6	F1(2): European–Spanish, Portuguese<br>F2(3): European<br>F3(3): European<br>F4(1): European<br>F5(1): European<br>F6(1): European
1	c.2T>G	Start-loss	1	European–German, Dutch, English

**CDH1* P/LP variant detected in AYA with relative with advanced diffuse gastric cancer

FN(n): where N is number of families in cohort with assigned *CDH1* P/LP variant and n is number of AYA individuals within that family. For example, there are two families with *CDH1* P/LP variant c.1587dup. Family #1 has three AYA variant carriers and family #2 has one AYA variant carrier.

Supplementary Table 2. Demographic information of AYA with advanced diffuse gastric cancer

Family #	AYA age at GC dx	AYA gastric cancer stage at dx	AYA race (Ethnicity)	AYA ancestry	# AYA relatives with advanced DGC	Age of advanced DGC dx in relative
1	28	Stage IV	White (Not Hispanic or Latino)	European-Swedish	1	58
2	32	Stage IV	Hispanic or Latino	Dominican Republic	0	-
3	33	Stage IV	White (Hispanic or Latino)	Mexican	1	47
4	33	Stage IB (T2N0M0)	White (Not Hispanic or Latino)	European	1	48

Abbreviations: AYA, adolescent and young adults; GC, gastric cancer; DGC, diffuse gastric cancer

Supplementary Table 3. Complications of prophylactic total gastrectomies performed at the National Institutes of Health.

**Complication**	**n=52**
Hospital re-admission ≤30 days, No. (%)	3 (6%)
Re-intervention ≤30 days, No. (%)	4 (8%)
Esophagojejunal anastomotic leak, No. (%)	3 (6%)
Esophagojejunal anastomotic stricture, No. (%)	2 (4%)
Incisional hernia repair, No. (%)	4 (8%)
Jejunal feeding tube placement, No. (%)	3 (6%)

Supplementary Table 4.

Univariable and multivariable logistic regression analyses with endoscopic surveillance versus prophylactic total gastrectomy (PTG) as outcome measure among adolescents and young adults (AYA) with germline *CDH1* P/LP variants in surveillance and PTG groups (n=171). Secondary analyses with endoscopic surveillance versus prophylactic total gastrectomy (PTG) as outcome measure among AYA with completed three-generation pedigree (n=101).

			**Univariable Analyses**			**Multivariable Analysis**		
		**N (%)**	**HR**	**95% CI**	***P* value**	**HR**	**95% CI**	***P* Value**
**Patient characteristics amongst Endoscopic surveillance (n=104) and Prophylactic total gastrectomy (n=67) groups**								
Age at enrollment, per year increase		171 (100)	1.04	0.99-1.10	0.11	1.07	0.99-1.14	0.07
Time Period of Enrollment	Early	40 (23.3)	Ref	-	-	Ref	-	-
	Mid	72 (42.1)	0.34	0.15-0.79	**0.012**	0.25	0.08-0.74	**0.012**
	Late	59 (34.5)	0.18	0.08-0.42	**<0.0001**	0.08	0.03-0.24	**<0.0001**
Sex	Female	119 (69.6)	Ref	-	-	Ref	-	-
	Male	52 (30.4)	0.59	0.30-1.18	0.14	0.58	0.24-1.40	0.23
Race/ Ethnicity	Non-Hispanic White	148 (86.6)	Ref	-	-	Ref	-	-
	Non-Hispanic Other	9 (5.3)	0.40	0.08-1.97	0.26	0.80	0.12-5.49	0.82
	Hispanic	14 (8.2)	0.38	0.10-1.41	0.15	0.49	0.08-2.92	0.44
Family History of Gastric Ca	Yes	146 (85.4)	Ref	-	-	Ref	-	-
	No	25 (14.6)	2.27	0.86-6.02	0.10	2.55	0.77-8.40	0.12
# of Family Members with GC, per additional member		146 (85.4)	1.06	0.95-1.20	0.29	-	-	-
Family History of Breast Ca	Yes	130 (76.0)	Ref	-	-	-	-	-
	No	40 (23.4)	1.35	0.66-2.77	0.41	-	-	-
	Unknown	1 (0.6)	-	-	-	-	-	-
Clinicopathologic Characteristics								

			Univariable Analyses			Multivariable Analysis		
		N (%)	HR	95% CI	*P* value	HR	95% CI	*P* Value
Signet Ring Cells on EGD	Absent	101 (59.1)	Ref	-	-	Ref	-	-
	Present	68 (39.8)	1.65	0.88-3.10	0.12	2.71	1.13-6.47	**0.025**
	Not assessed	2 (1.2)	-	-	-	-	-	-
Number of EGDs Performed	0	2 (1.2)	-	-	-	-	-	-
	1	103 (60.2)	Ref	-	-	Ref	-	-
	2	34 (19.9)	0.56	0.25-1.24	0.15	0.32	0.11-0.91	**0.032**
	≥3	32 (18.7)	0.07	0.02-0.30	**0.0004**	0.03	0.01-0.14	**<0.0001**
**Patient characteristics amongst AYA with three-generation pedigree**								
Age at enrollment, per year increase		101 (100)	1.05	0.98-1.12	0.16	1.07	0.98-1.17	0.11
Time Period of Enrollment	Early	21 (20.8)	Ref	-	-	Ref	-	-
	Mid	36 (35.6)	0.32	0.10-1.01	0.053	0.24	0.05-1.13	0.071
	Late	44 (43.6)	0.21	0.07-0.64	**0.0064**	0.09	0.02-0.41	**0.0019**
Sex	Female	70 (69.3)	Ref	-	-	Ref	-	-
	Male	31 (30.7)	0.67	0.28-1.58	0.36	0.55	0.18-1.66	0.29
Race/Ethnicity	Non-Hispanic White	90 (89.1)	Ref	-	-	Ref	-	-
	Non-Hispanic Other	5 (5.0)	0.29	0.03-2.66	0.27	0.84	0.06-11.6	0.90
	Hispanic	6 (5.9)	0.76	0.12-4.78	0.77	1.70	0.12-23.6	0.69
Family History of Gastric Ca	Yes	101 (100)	-	-	-	-	-	-
	No	0	-	-	-	-	-	-
# of Family Members with GC, per additional member		101 (100)	1.08	0.92-1.27	0.35	-	-	-
Family History of Breast Ca	Yes	73 (72.3)	Ref	-	-	-	-	-
	No	28 (27.7)	1.28	0.54-3.07	0.58	-	-	-
Clinicopathologic Characteristics								
Signet Ring Cells on EGD	Absent	53 (52.5)	Ref	-	-	Ref	-	-
	Present	45 (44.6)	1.74	0.78-3.89	0.18	3.34	1.10-10.1	**0.033**
	Not assessed	3 (3.0)	-	-	-	-	-	-

			Univariable Analyses			Multivariable Analysis		
		**N (%)**	**HR**	**95% CI**	***P* value**	**HR**	**95% CI**	***P* Value**
Number of EGDs Performed	0	3 (3.0)	-	-	-	-	-	-
	1	61 (60.4)	Ref	-	-	Ref	-	-
	2	22 (21.8)	0.66	0.25-1.76	0.41	0.57	0.18-1.86	0.35
	≥3	15 (14.9)	0.06	0.01-0.46	**0.0071**	0.02	0.01-0.22	**0.0012**
Highest Gastric Cancer Stage of Relative	T1a	18 (17.8)	Ref	-	-	Ref	-	-
	>T1a	83 (82.2)	1.86	0.64-5.43	0.26	1.21	0.27-5.47	0.81
Relative Underwent TG	Yes	15 (14.9)	Ref	-	-	Ref	-	-
	No	86 (85.1)	1.30	0.43-3.98	0.64	0.89	0.19-4.25	0.88

Abbreviations: HR, hazards ratio; CI, confidence interval; GC, gastric cancer.

Supplementary Figures

Supplementary Figure 1. Bivariate analysis of age of proband diagnosis of signet ring cells on upper endoscopy and age of relative cancer diagnosis.

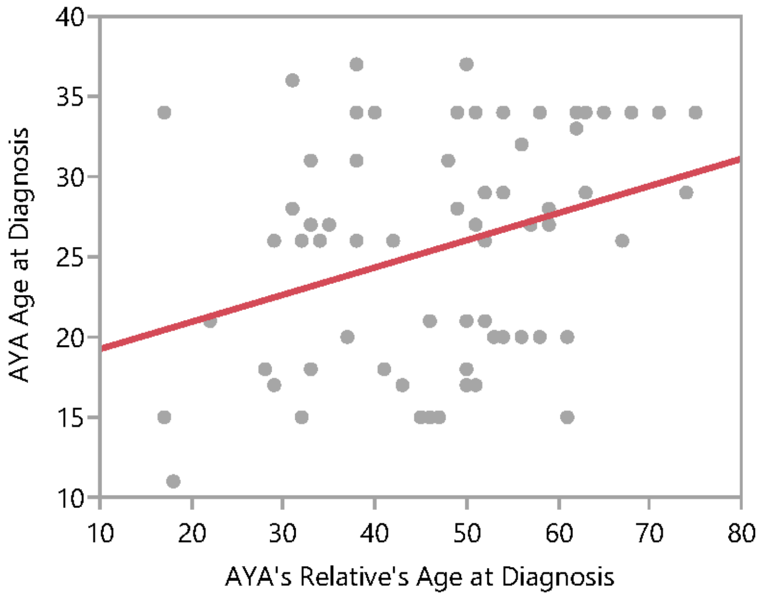

Supplement: djaf002_Supplementary_Data [file djaf002_supplementary_data.pdf]
